# Burden of fatigue or pruritus in patients with primary biliary cholangitis: a United States matched case–control study

**DOI:** 10.57264/cer-2025-0179

**Published:** 2026-08-12

**Authors:** Sonal Kumar, Nisreen Shamseddine, Hongbo Yang, Su Zhang, Jingyi Chen, Kris V Kowdley

**Affiliations:** 1 Weill Cornell Medical College, Cornell University, New York, NY 10065, US; 2 Ipsen, Cambridge, MA 02142, US; 3 Analysis Group, Inc., Boston, MA 02199, US; 4 Liver Institute Northwest, Seattle, WA 98105, US

**Keywords:** comorbidity burden, fatigue, primary biliary cholangitis, pruritus, rare disease, real-world data

## Abstract

**Aim:**

Primary biliary cholangitis (PBC) is a rare liver disease associated with high morbidity. This study assessed the burden of fatigue and/or pruritus among patients with PBC in the US.

**Materials & methods:**

This retrospective study used IQVIA PharMetrics^®^ Plus data (2016–2022). Patients with PBC and fatigue and/or pruritus were selected as cases. Controls were patients with PBC (no fatigue nor pruritus), matched 1:1 to cases by key characteristics. The index date for cases was a random symptom diagnosis date post-initial PBC diagnosis and for controls, a random medical visit date matching the time distribution from initial PBC diagnosis to index. Cumulative incidence of PBC comorbidities was described using Kaplan–Meier analysis and compared via Cox Proportional hazard models. Generalized estimating equations compared healthcare resource use (HRU) and costs per-patient-per-year.

**Results:**

A total of 1839 fatigue cases/controls (mean age [years]: 56.5; 88.7% female) and 760 pruritus cases/controls were included (mean age [years]: 55.8; 90.8% female). Comorbidities at 1, 3 and 5-years post-index were higher for cases than controls (fatigue: 1.7 vs 0.7, 2.2 vs 0.9 and 2.5 vs 1.0; pruritus: 1.9 vs 0.8, 2.3 vs 1.0 and 2.7 vs 1.0; all p < 0.001). Common comorbidities were anxiety, urinary tract infection, depression and sleep disorders (hazard ratios in cases vs controls: fatigue, 1.3–4.0; pruritus, 1.5–2.8; all p < 0.01). One-year post-index, cases had higher rates of healthcare visits (incidence rate ratios: fatigue, 1.8–5.8; pruritus 1.6–6.1) and total healthcare costs (mean cost difference: fatigue, $42,515; pruritus $40,536).

**Conclusion:**

Patients with PBC who experience fatigue and/or pruritus faced a greater clinical and economic burden compared with those without these symptoms, highlighting the need for effective treatments to alleviate PBC symptoms.

## Plain language summary: How fatigue or itching affect people with primary biliary cholangitis in the US compared with people without the disease

**What is this article about?:**

Primary biliary cholangitis (PBC) is a long-term rare liver disease that can increase the risk of developing other health problems. PBC symptoms can make it difficult for patients to have a good quality of life. The most common symptoms of PBC are tiredness (fatigue) and itchiness (pruritus).

**What were the results?:**

This study found that patients with PBC who experienced tiredness or itchiness were more likely to have additional health problems over time than patients who were not itchy or tired. These health problems were: increased risk of anxiety, urinary tract infections, depression and trouble sleeping. In addition, patients with tiredness or itchiness had about two to six-times more visits to the hospital, doctor’s office or emergency room than patients without these symptoms. On average, the total healthcare costs were more than $40,000 higher among patients with tiredness or itchiness than those without, mostly because of more hospital stays, showing that tiredness and itchiness can increase the financial burden experienced by patients.

**Why is this important?:**

These results show the importance of finding effective treatments to reduce the symptoms of tiredness or itchiness for people living with PBC.

Primary biliary cholangitis (PBC) is a rare, chronic, progressive, cholestatic autoimmune liver disease, with an estimated prevalence of 1.9–40.2 per 100,000 people worldwide, which has shown an upward trend over time [1–3]. PBC is most commonly diagnosed in people aged 40–60 years and predominantly affects women [4]. Fatigue and pruritus are the most common symptoms of PBC, experienced by up to an estimated 75% of patients, and can have a considerable impact on patients’ quality of life [5–7].

To date, the US Food and Drug Administration (FDA) has approved four treatments for PBC: ursodeoxycholic acid (UDCA), obeticholic acid (OCA, marketed as OCALIVA^®^; OCA received accelerated approval in 2016, the FDA recommended against full approval in 2024, and OCA was voluntarily withdrawn from the US market following a request from the FDA in 2025), and the recently conditionally approved proliferator-activated receptor (PPAR) agonists elafibranor (PPARα/δ agonist) and seladelpar (PPARδ agonist) [8–14]. UDCA is the standard first-line treatment for PBC and has been shown to improve prognosis and reduce mortality, but it does not alleviate fatigue or pruritus [8,15,16]. For second-line therapy, elafibranor, seladelpar, and formerly OCA are each approved in combination with UDCA for patients with PBC who have an inadequate response to UDCA alone, or as monotherapy for those unable to tolerate UDCA [10–12]. However, fatigue and pruritus were common adverse effects reported in OCA trials [17], and severe pruritus had been included as a warning on the OCA US product label [10]. In contrast, elafibranor and seladelpar have been shown to potentially reduce pruritus in clinical trials [18,19].

Prior studies have documented the high comorbidity burden associated with PBC [7,20,21], and US-specific studies have reported substantial healthcare resource use (HRU) and healthcare costs incurred by patients with PBC in specific populations (e.g., inpatient [IP] populations, Medicare beneficiaries) [22,23]. However, comprehensive assessments of the clinical and economic burden associated with specific symptoms of PBC have been scarce, with only one US claims analysis reporting higher prevalence of comorbidities among patients with PBC who experienced pruritus relative to those without PBC or pruritus [20]. To understand the incremental burden of fatigue and/or pruritus, this study evaluated the comorbidity burden, HRU and healthcare costs associated with these symptoms among patients with PBC in the US.

## Materials & methods

### Study design, data source & cohort creation

This retrospective, matched case–control study used claims data from the IQVIA PharMetrics^®^ Plus database (January 2016 to December 2022). The database contains integrated medical and pharmacy claims data of over 215 million unique enrollees across the US since 2006. As this study used de-identified claims data, no institutional review board (IRB) ethical review was required.

Adult patients with a PBC diagnosis on or after 1 January 2016, were identified based on International Classification of Diseases, 9th/10th Revision, Clinical Modification codes (ICD-9-CM code 571.6 or ICD-10-CM code K743). Two case cohorts (fatigue and pruritus) and two respective matched control cohorts were created. Fatigue cases comprised patients with PBC who had a diagnosis of fatigue (ICD codes R53.8x and R53.1) and pruritus cases comprised patients with PBC who had a diagnosis of pruritus (ICD code L29.x). The diagnosis of fatigue or pruritus occurred after the initial PBC diagnosis captured within the study period. The pruritus and fatigue case cohorts were not mutually exclusive; the presence of both symptoms did not impact patient eligibility for inclusion in either cohort. Fatigue or pruritus controls comprised patients with PBC who had no diagnosis of fatigue or pruritus any time following diagnosis of PBC.

A combination of propensity score and exact matching was used to create a 1:1 ratio of cases and matched controls with balanced baseline characteristics and a reasonable sample size. Applying exact matching to all variables would have resulted in a small sample size; therefore, exact matching was applied to sex, insurance type and geographic region while propensity score matching was applied to age at index, baseline period PBC duration and length of follow-up duration.

**Figure 1.**
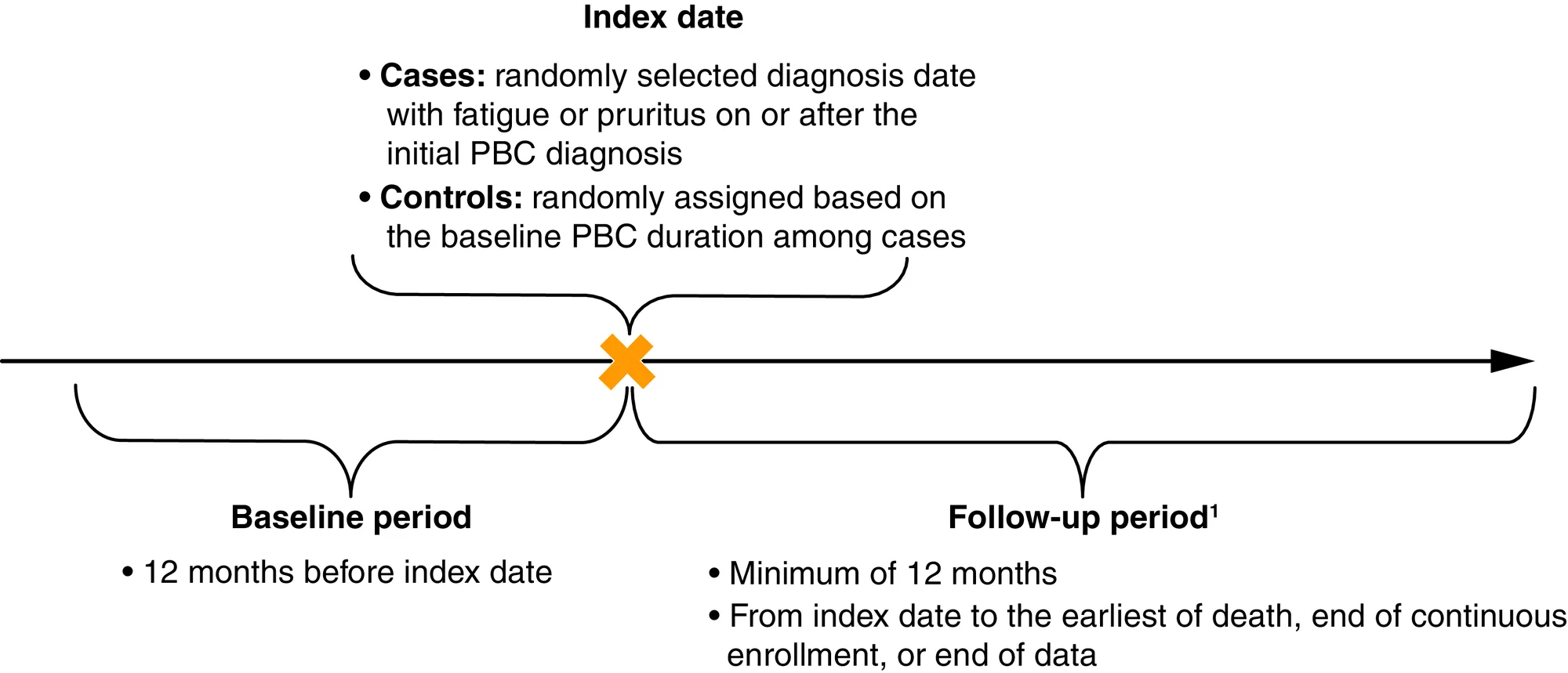
Study design diagram. Timeline of the baseline period and follow-up period relative to the index date. ^1^Cumulative comorbidity burden was assessed until the end of the follow-up period; HRU and costs were assessed during the 1-year post-index. HRU: Healthcare resource use; PBC: Primary biliary cholangitis.

A study design diagram is presented in Figure 1. For the fatigue or pruritus cases, the index date was defined as a random date for diagnosis after the initial PBC diagnosis. Using a random date for diagnosis is a common approach in disease burden studies, especially for chronic conditions, as it captures both incident and prevalent patients, therefore better representing the real-world disease burden and patient population [24–26]. For controls, the index date was defined as a random medical visit date that allowed the distribution of time from initial PBC diagnosis to the index date to match the time distribution in cases. For both cases and controls, the baseline period was defined as the 12 months before the index date, and the follow-up period (with a minimum of 12 months) spanned from the index date to the earliest date of death, end of continuous enrollment, or end of data collection.

### Study outcomes

The study outcomes included comorbidity burden and all-cause HRU and healthcare costs, assessed separately for the fatigue or pruritus cohorts.

Comorbidity burden during the baseline and follow-up periods was described and compared between cases and matched controls. Comorbidities in this study were selected based on the literature [20,22,23] and clinician input. For each selected comorbidity, the cumulative incidence rates by 1, 3 and 5-years post-index were assessed. The study inclusion criteria allowed patients to have these selected comorbidities present in the baseline period. For the pruritus cohort, pruritus was not considered a comorbidity; for the fatigue cohort, fatigue was not considered a comorbidity. The median number of observed comorbidities in cases and matched controls ranged from one to three between years one and five. As such, it was deemed appropriate to assess the mean number of comorbidities per patient with continuous enrollment and the proportions of patients with at least one, three, and five comorbidities by 1, 3 and 5-years post-index. No additional adjustment was applied on baseline comorbidity profiles. The number of cumulative comorbidities patients experienced at each timepoint were reported and compared, including those comorbidities developed before index date.

All-cause HRU and healthcare costs were summarized on a per-patient-per-year (PPPY) basis during the 1-year post-index period only, and compared between cases and matched controls. HRU outcomes included the number of IP admissions, the length of stay for IP admission, the number of outpatient (OP) visits and the number of emergency room (ER) visits. Total healthcare costs were defined as the sum of medical costs and pharmacy costs. Medical costs included costs of IP admission, OP visits, ER visits and other medical services (including durable medical equipment and dental or visual care). Pharmacy costs included expenses for PBC medications and other medications. The latter included pharmacy costs beyond PBC-related and pruritus-related (pruritus cohort) or fatigue-related medications (fatigue cohort). All costs were measured as total payments from payers and were adjusted to 2023 US dollar value.

### Statistical analysis

Key patient characteristics and comorbidities during the baseline period were summarized using means, medians, standard deviations (SDs) and interquartile range (IQR) for continuous variables, and frequency counts and percentages for categorical variables. Standardized mean differences (SMDs) were used to assess balance between cases and matched controls, with SMDs ≥0.1 considered as imbalanced between the matched pairs [27].

Wilcoxon signed-rank tests were used to compare the mean number of comorbidities per patient. McNemar tests were used to compare the proportion of patients with one, three, and five comorbidities between cases and controls during the baseline and follow-up periods. Kaplan–Meier (KM) analysis was used to describe the cumulative incidence of each selected comorbidity during follow-up and the hazard ratio (HR) of developing the comorbidity in cases versus (vs) controls was estimated. All analyses in this study were based on post-matching cohorts (i.e., comparison after matching).

HRU and healthcare costs were compared using generalized estimating equations (GEEs) to account for the correlation between cases and controls due to matching. For HRU, odds ratios (ORs) were calculated using a binomial distribution and logit-link function, and incidence rate ratios (IRRs) were calculated using a negative binomial distribution and log-link function. For healthcare costs, GEE models with a Tweedie distribution and a log-link function were used and adjusted mean cost differences were estimated. These models were employed as they are commonly used in health economic analysis for outcomes with skewed and/or overdispersed data, such as HRU and healthcare costs.

All statistical analyses were conducted using R (version 3.6.2).

## Results

### Sample size

**Figure 2.**
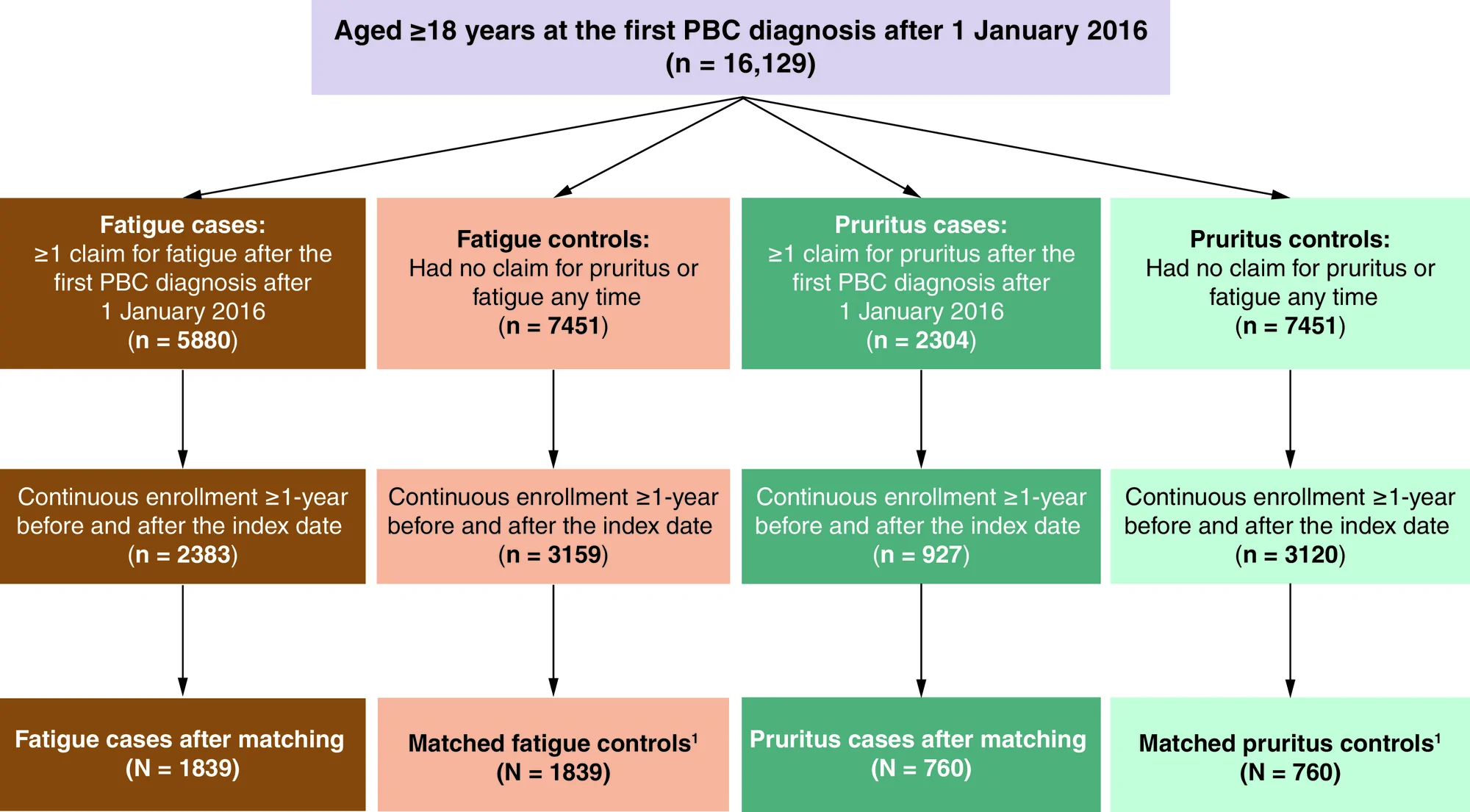
Sample selection and matching. Flow diagram with cohort inclusions and matching; includes sample sizes. ^1^Controls were matched to respective cases at a 1:1 ratio by age at index, sex, insurance type, geographic region, duration from the initial PBC diagnosis to the index date and length of follow-up period, using exact and propensity score matching. PBC: Primary biliary cholangitis.

After matching, 1839 fatigue cases and matched controls and 760 pruritus cases and matched controls were included in the analyses. The median follow-up duration was 21.5 and 23.3 months for the matched fatigue cohorts and matched pruritus cohorts, respectively (for full sample selection see Figure 2).

### Baseline characteristics

Baseline patient characteristics for the fatigue or pruritus cases and controls, before matching, are presented in Supplementary Table 1.

After exact and propensity score matching (described in Methods), patient demographic characteristics were similar between cases and their respective matched controls (Table 1). The mean (SD) age was 56.4 (10.7) and 56.7 (10.1) years for fatigue cases and matched controls (SMD = 0.032), respectively, and 55.6 (10.7) and 56.0 (9.9) years for pruritus cases and matched controls (SMD = 0.038), respectively. In both cohorts, most patients were female (fatigue cohorts: 88.7%; pruritus cohorts: 90.8%); the geographic region from which most patients were enrolled was the South (fatigue cohorts: 44.8%; pruritus cohorts: 50.5%), followed by the Midwest (fatigue cohorts: 26.2%; pruritus cohorts: 23.0%); and over 90% of patients were covered by commercial insurance (fatigue cohorts: 91.6%; pruritus cohorts: 92.5%).

**Table 1.** Baseline characteristics of post-matched fatigue or pruritus cohorts.

	Fatigue cohorts			Pruritus cohorts		
	Case (N = 1839)	Control (N = 1839)	SMD^†^	Case (N = 760)	Control (N = 760)	SMD^†^
**Demographic characteristics**						
Age at index (years), mean ± SD	56.4 ± 10.7	56.7 ± 10.1	0.032	55.6 ± 10.7	56.0 ± 9.9	0.038
Median	57.0	58.0		56.0	57.0	
IQR	(50.0, 62.0)	(51.0, 63.0)		(49.0, 62.0)	(50.0, 62.0)	
Female, n (%)	1631 (88.7%)	1631 (88.7%)	0.000	690 (90.8%)	690 (90.8%)	0.000
Geographic region, n (%)			0.000			0.000
South	823 (44.8%)	823 (44.8%)		384 (50.5%)	384 (50.5%)	
Midwest	482 (26.2%)	482 (26.2%)		175 (23.0%)	175 (23.0%)	
Northeast	306 (16.6%)	306 (16.6%)		122 (16.1%)	122 (16.1%)	
West	227 (12.3%)	227 (12.3%)		79 (10.4%)	79 (10.4%)	
Unknown	1 (0.1%)	1 (0.1%)		0 (0.0%)	0 (0.0%)	
Insurance type			0.000			0.000
Commercial	1,685 (91.6%)	1,685 (91.6%)		703 (92.5%)	703 (92.5%)	
Medicare Advantage	154 (8.4%)	154 (8.4%)		57 (7.5%)	57 (7.5%)	
**PBC disease characteristics**						
First observed diagnosis^‡^, n (%)			0.072			0.075
2016	817 (44.4%)	819 (44.5%)		365 (48.0%)	346 (45.5%)	
2017	292 (15.9%)	316 (17.2%)		132 (17.4%)	143 (18.8%)	
2018	231 (12.6%)	228 (12.4%)		89 (11.7%)	93 (12.2%)	
2019	243 (13.2%)	206 (11.2%)		84 (11.1%)	81 (10.7%)	
2020	192 (10.4%)	196 (10.7%)		70 (9.2%)	70 (9.2%)	
2021	64 (3.5%)	74 (4.0%)		20 (2.6%)	27 (3.6%)	
PBC duration^§^ (months), mean ± SD	19.3 ± 16.4	19.0 ± 16.1	0.019	21.0 ± 16.8	21.3 ± 16.9	0.015
Median	14.8	14.4		16.2	16.4	
IQR	(6.5, 27.5)	(6.4, 27.5)		(8.4, 31.1)	(8.1, 31.1)	
**Comorbidities**						
CCI^¶^, mean ± SD	3.2 ± 1.9	2.6 ± 1.3	0.391	3.0 ± 1.7	2.5 ± 1.2	0.347
Median	3.0	2.0		2.0	2.0	
IQR	(2.0, 4.0)	(2.0, 3.0)		(2.0, 4.0)	(2.0, 3.0)	
**Comorbid liver complications, n (%)**						
Cirrhosis	529 (28.8%)	322 (17.5%)	0.269	212 (27.9%)	137 (18.0%)	0.236
Abnormal liver test	421 (22.9%)	345 (18.8%)	0.102	184 (24.2%)	140 (18.4%)	0.142
Autoimmune hepatitis	214 (11.6%)	179 (9.7%)	0.062	95 (12.5%)	71 (9.3%)	0.101
Portal hypertension	209 (11.4%)	96 (5.2%)	0.224	95 (12.5%)	46 (6.1%)	0.224
Primary sclerosing cholangitis	97 (5.3%)	47 (2.6%)	0.141	45 (5.9%)	16 (2.1%)	0.195
Chronic hepatitis NOS	44 (2.4%)	49 (2.7%)	0.017	20 (2.6%)	16 (2.1%)	0.035
Liver fibrosis	37 (2.0%)	26 (1.4%)	0.046	18 (2.4%)	14 (1.8%)	0.037
Cholecystitis	41 (2.2%)	19 (1.0%)	0.095	11 (1.4%)	5 (0.7%)	0.077
**Additional comorbidities/PBC symptoms, n (%)**						
Pruritus	179 (9.7%)	0 (0.0%)	0.464	276 (36.3%)	0 (0.0%)	1.068
Fatigue	647 (35.2%)	0 (0.0%)	1.042	186 (24.5%)	0 (0.0%)	0.805
Dyslipidemia	842 (45.8%)	711 (38.7%)	0.145	329 (43.3%)	310 (40.8%)	0.051
Hypertension	834 (45.4%)	674 (36.7%)	0.178	322 (42.4%)	278 (36.6%)	0.119
Anxiety	439 (23.9%)	256 (13.9%)	0.256	160 (21.1%)	112 (14.7%)	0.165
Type 2 diabetes	363 (19.7%)	268 (14.6%)	0.137	129 (17.0%)	95 (12.5%)	0.126
Depression	396 (21.5%)	197 (10.7%)	0.297	140 (18.4%)	81 (10.7%)	0.222
Osteoporosis	216 (11.7%)	165 (9.0%)	0.091	98 (12.9%)	63 (8.3%)	0.150
Urinary tract infection	286 (15.6%)	140 (7.6%)	0.250	96 (12.6%)	58 (7.6%)	0.166
Gallstone disease (cholelithiasis)	177 (9.6%)	121 (6.6%)	0.112	67 (8.8%)	39 (5.1%)	0.145
Diarrhea	248 (13.5%)	103 (5.6%)	0.271	106 (13.9%)	29 (3.8%)	0.362
Sleep-related issues	188 (10.2%)	74 (4.0%)	0.243	63 (8.3%)	30 (3.9%)	0.182
Inflammatory bowel disease	151 (8.2%)	62 (3.4%)	0.208	61 (8.0%)	27 (3.6%)	0.192
Rheumatoid arthritis	138 (7.5%)	64 (3.5%)	0.177	56 (7.4%)	15 (2.0%)	0.258
Ulcerative colitis	99 (5.4%)	56 (3.0%)	0.117	46 (6.1%)	18 (2.4%)	0.184
Sjögren’s syndrome	120 (6.5%)	60 (3.3%)	0.152	46 (6.1%)	25 (3.3%)	0.131
Raynaud’s syndrome	77 (4.2%)	53 (2.9%)	0.071	27 (3.6%)	16 (2.1%)	0.087
Systemic lupus erythematosus	60 (3.3%)	33 (1.8%)	0.094	29 (3.8%)	8 (1.1%)	0.180
Crohn’s disease	59 (3.2%)	30 (1.6%)	0.103	19 (2.5%)	11 (1.4%)	0.076
Cognitive impairment	72 (3.9%)	18 (1.0%)	0.191	25 (3.3%)	9 (1.2%)	0.143
Autoimmune thyroid disease	59 (3.2%)	28 (1.5%)	0.111	16 (2.1%)	8 (1.1%)	0.085

† SMD ≥0.1 was considered as imbalanced between the matched pairs [27].

‡ Year of first use of PBC diagnostic code post-2016.

§ Baseline PBC duration is measured from the first observed PBC diagnosis to the index date.

¶ CCI was calculated based on Quan *et al.* 2011 [28].

CCI: Charlson Comorbidity Index; IQR: Interquartile range; NOS: Not otherwise specified; PBC: Primary biliary cholangitis; SD: Standard deviation; SMD: Standardized mean difference.

### Comorbidity burden

**Figure 3.**
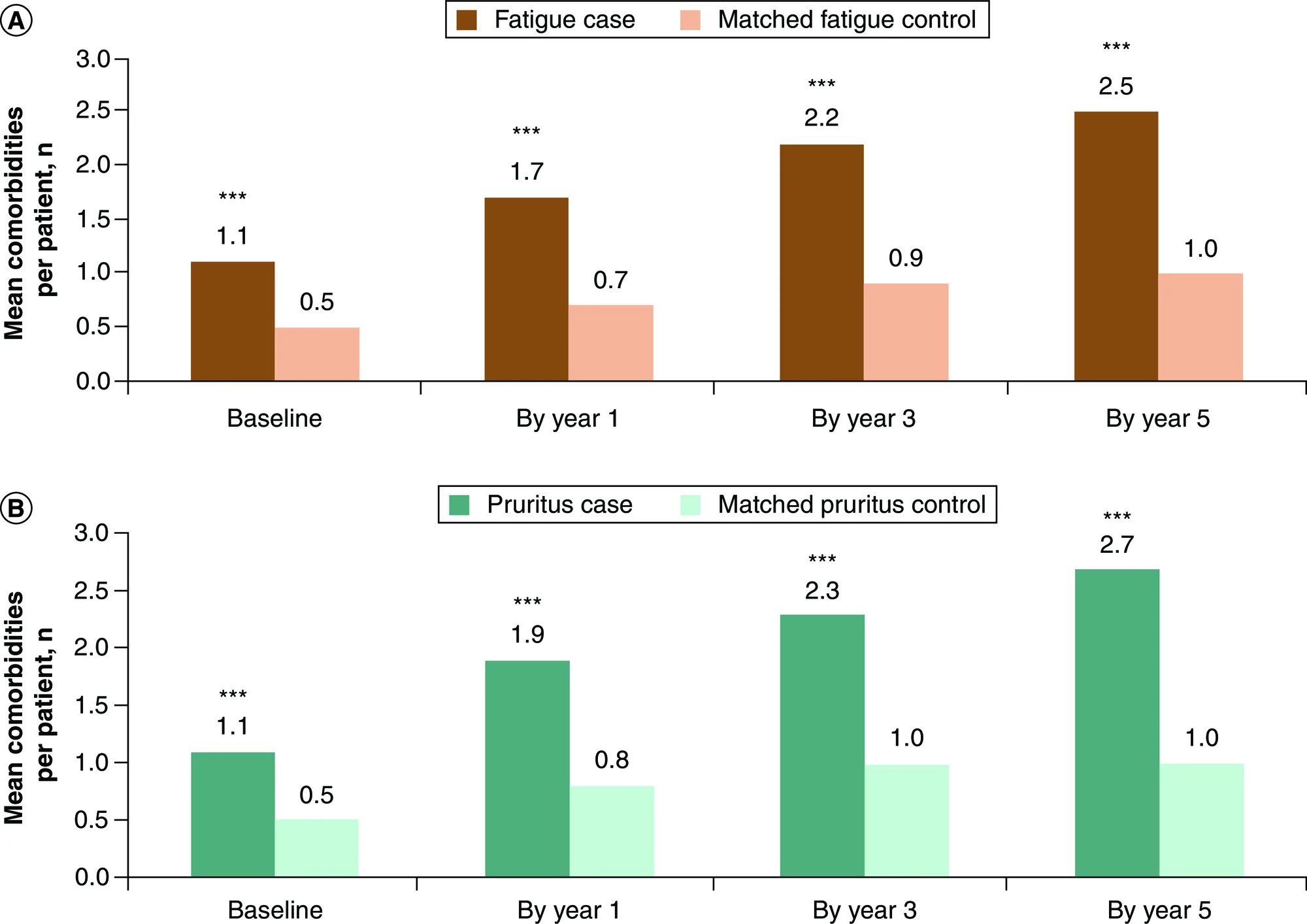
Number of comorbidities per patient among fatigue or pruritus cohorts during baseline and by post-index year. **(A)** Fatigue cohorts. **(B)** Pruritus cohorts. The mean number of comorbidities per patient in **(A)** the fatigue cohort and matched controls and **(B)** the pruritus cohort and matched controls at baseline and by 1, 3 and 5-years post-index for patients with continuous enrollment to that year. Wilcoxon signed-rank tests were used to compare the mean number of comorbidities per patient. *** indicates p < 0.001 for cases versus controls; significance level: p < 0.05.

Baseline comorbidity burden was higher among cases than matched controls in both cohorts. The mean Charlson Comorbidity Index (CCI) was 3.2 for fatigue cases vs 2.6 for matched controls (SMD = 0.391) and 3.0 for pruritus cases vs 2.5 for matched controls (SMD = 0.347; Table 1). For the fatigue or pruritus cohorts, the mean number of comorbidities per patient was 1.1 at index date for cases vs 0.5 for matched controls (both p < 0.001) (Figure 3). Common comorbid liver complications at baseline across all cohorts included cirrhosis (17.5–28.8%), an abnormal liver test (18.4–24.2%), autoimmune hepatitis (9.3–12.5%) and portal hypertension (5.2–12.5%). Other common comorbidities across all cohorts included dyslipidemia (38.7–45.8%), hypertension (36.6–45.4%) and anxiety (13.9–23.9%) (Table 1). During the follow-up period, the number of comorbidities was significantly higher among cases than matched controls at all timepoints assessed. For the fatigue cohorts, the number of comorbidities during the follow-up period was higher in cases vs matched controls: year one post-index, 1.7 versus 0.7; year three, 2.2 versus 0.9; year five, 2.5 vs 1.0 (all p < 0.001). For the pruritus cohorts, these were also higher in cases vs matched controls: year one post-index, 1.9 vs 0.8; year three, 2.3 vs 1.0; year five, 2.7 vs 1.0 (all p < 0.001; Figure 3). A significantly higher proportion of cases had at least one, three or five unique comorbidities than matched controls by 1-year post-index; this trend continued by 3 and 5-years post-index (Table 2).

**Table 2.** Number of comorbidities per patient among fatigue and pruritus cohorts during baseline and by post-index year.

	Comorbidities per patient, n (%)^†^		
	1+	3+	5+
(A) Fatigue cohorts			
**Baseline**			
Case (N = 1839)	1108 (60.3%)	238 (12.9%)	21 (1.1%)
Control (N = 1839)	642 (34.9%)	48 (2.6%)	2 (0.1%)
p-value^‡^	<0.001	<0.001	<0.001
**By year 1**			
Case (N = 1839)	1382 (75.1%)	488 (26.5%)	76 (4.1%)
Control (N = 1839)	849 (46.2%)	126 (6.9%)	6 (0.3%)
p-value^‡^	<0.001	<0.001	<0.001
**By year 3**			
Case (N = 363)	302 (83.2%)	140 (38.6%)	35 (9.6%)
Control (N = 363)	192 (52.9%)	35 (9.6%)	0 (0.0%)
p-value^‡^	<0.001	<0.001	<0.001
**By year 5**			
Case (N = 69)	59 (85.5%)	33 (47.8%)	8 (11.6%)
Control (N = 69)	45 (65.2%)	6 (8.7%)	0 (0.0%)
p-value^‡^	<0.05	<0.001	<0.05
**(B) Pruritus cohorts**			
**Baseline**			
Case (N = 760)	449 (59.1%)	104 (13.7%)	17 (2.2%)
Control (N = 760)	256 (33.7%)	20 (2.6%)	1 (0.1%)
p-value^‡^	<0.001	<0.001	<0.001
**By year 1**			
Case (N = 760)	600 (78.9%)	231 (30.4%)	54 (7.1%)
Control (N = 760)	364 (47.9%)	57 (7.5%)	3 (0.4%)
p-value^‡^	<0.001	<0.001	<0.001
**By year 3**			
Case (N = 183)	158 (86.3%)	72 (39.3%)	20 (10.9%)
Control (N = 183)	109 (59.6%)	23 (12.6%)	1 (0.5%)
p-value^‡^	<0.001	<0.001	<0.001
**By year 5**			
Case (N = 25)	24 (96.0%)	13 (52.0%)	4 (16.0%)
Control (N = 25)	15 (60.0%)	2 (8.0%)	0 (0.0%)
p-value^‡^	<0.05	<0.001	0.1

† The average number of comorbidities per patient by 1, 3 and 5-years post-index were described among matched pairs with continuous enrollment to that year.

‡ Values of p were calculated using Wilcoxon signed-rank tests for continuous variables and McNemar tests for binary variables.

**Figure 4.**
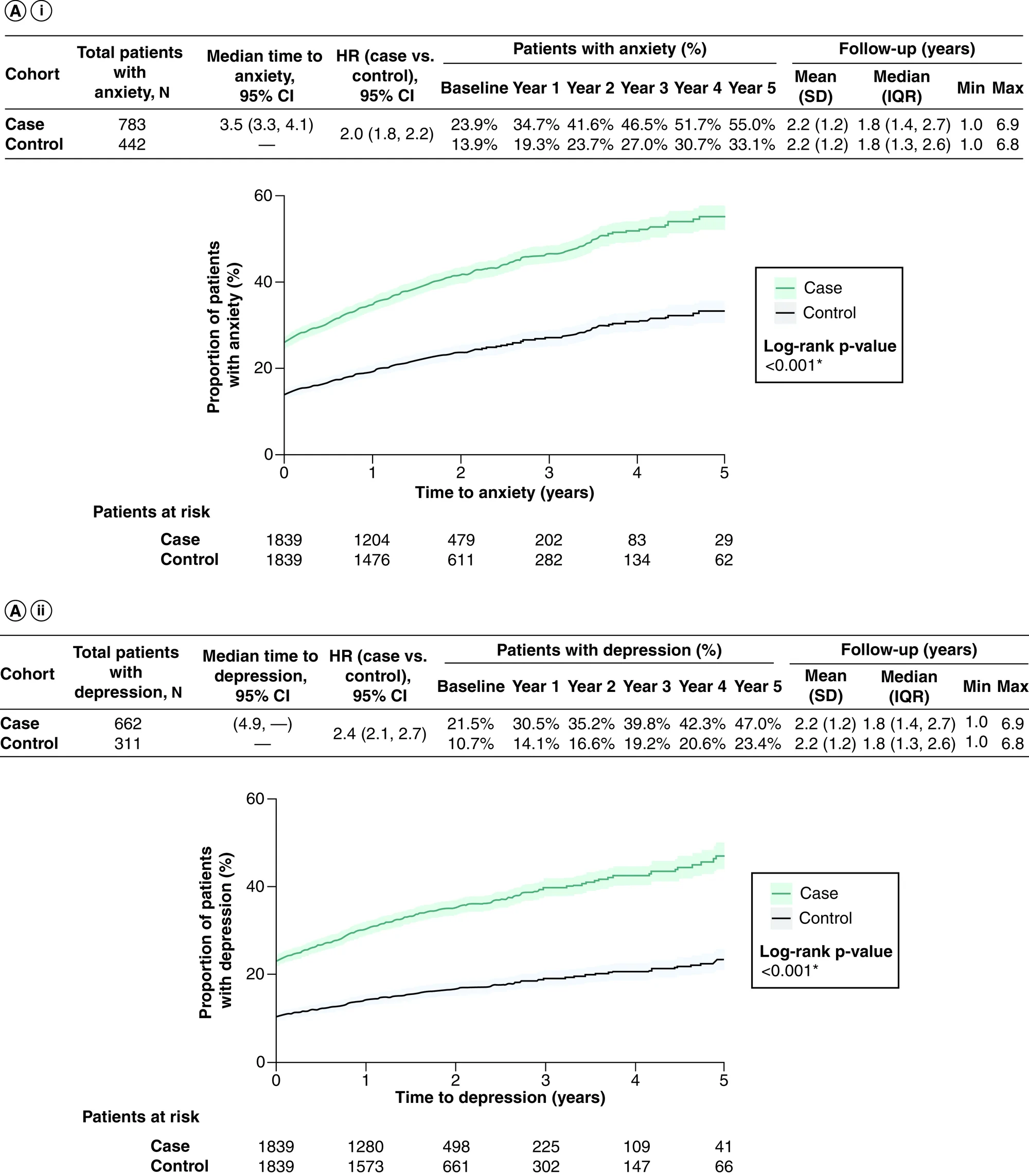

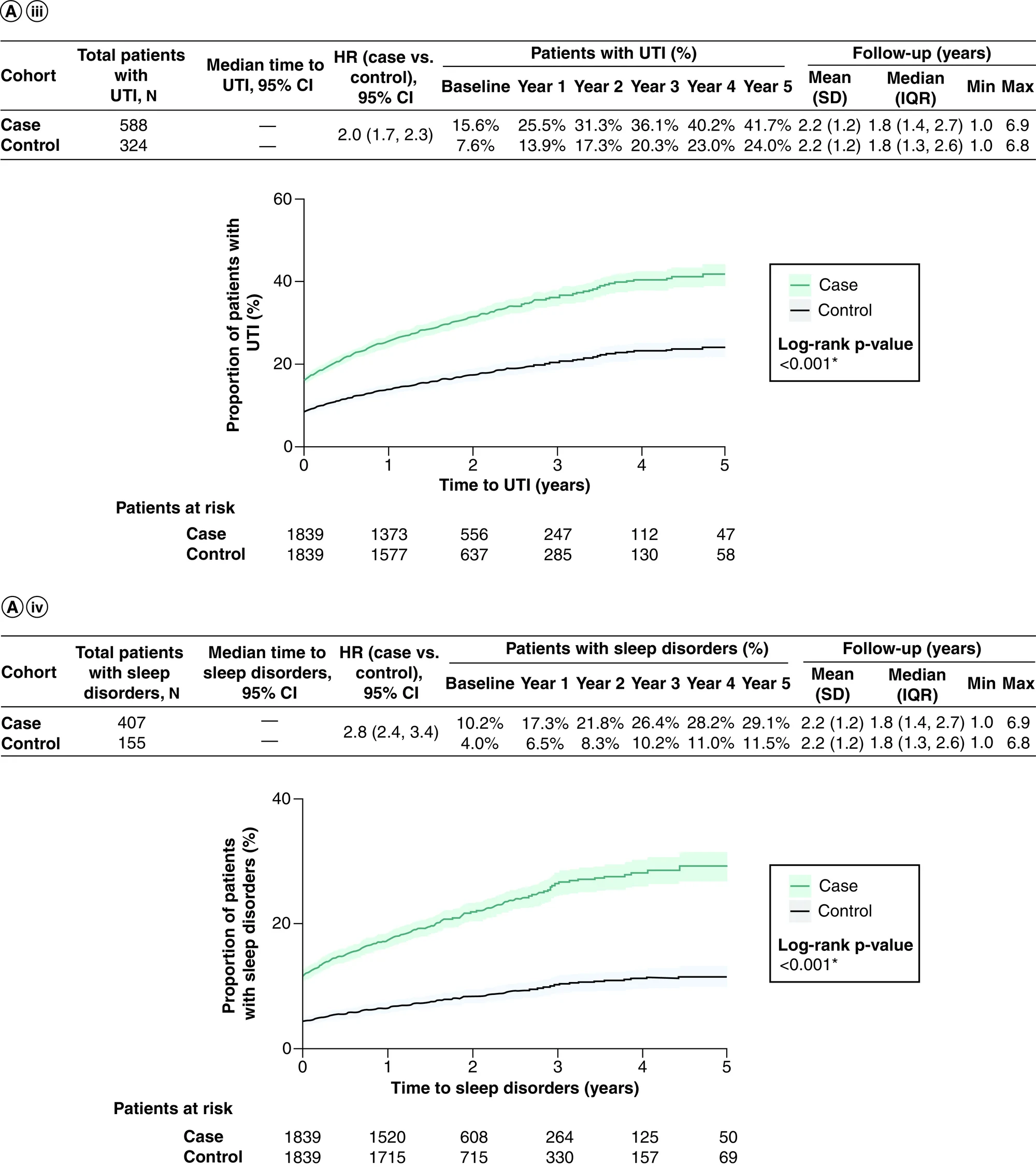

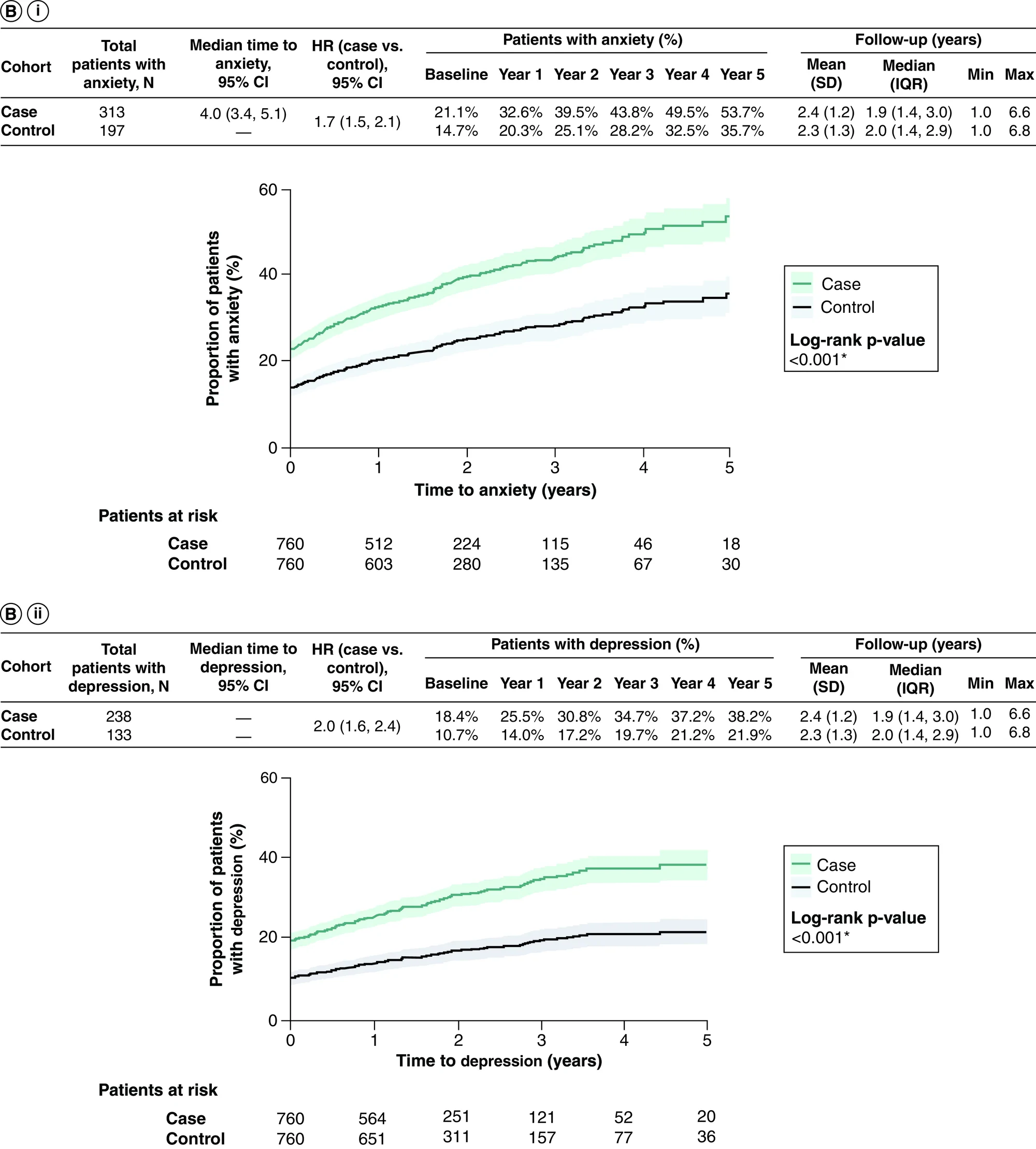

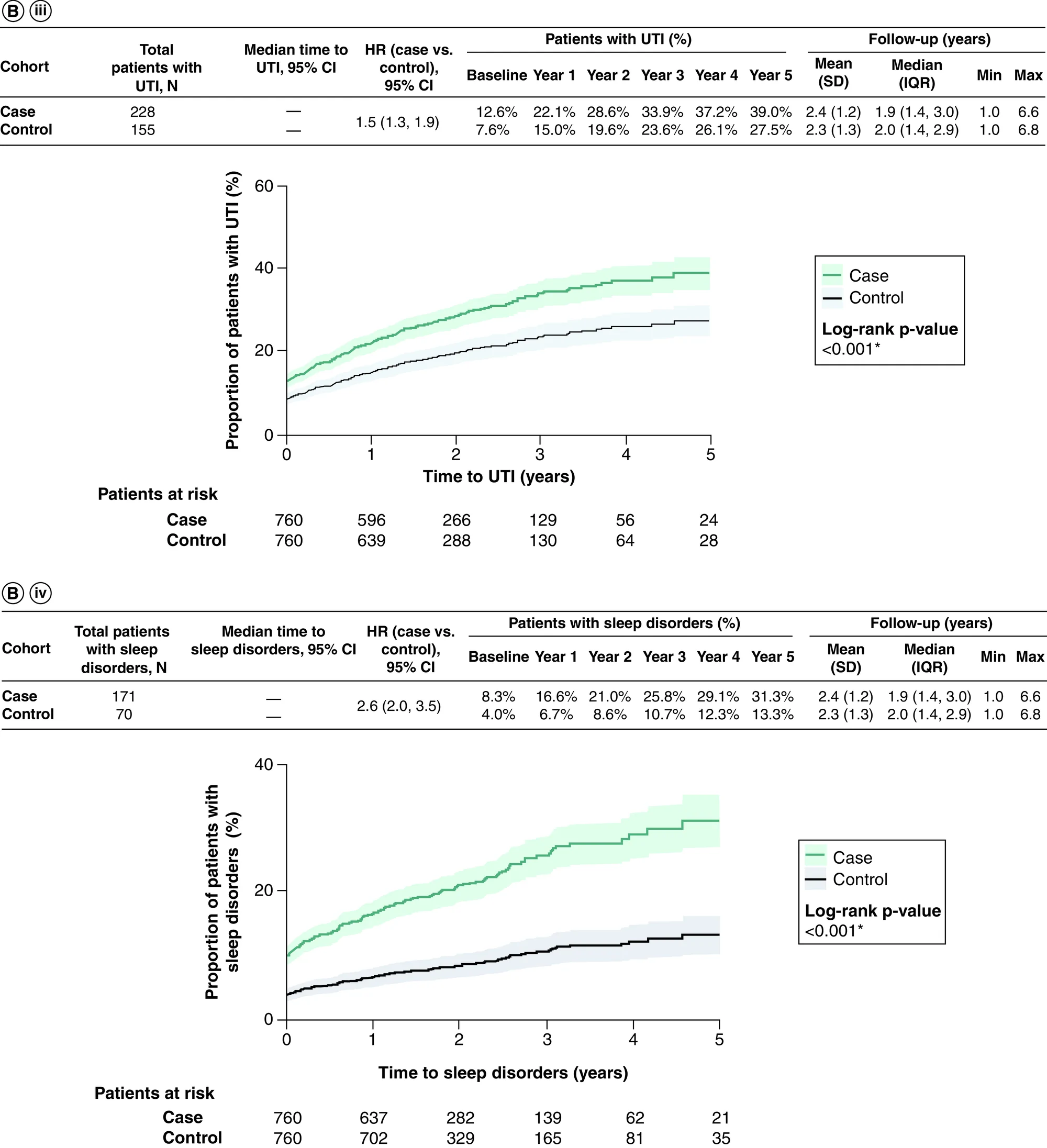
Cumulative incidence of the most common comorbidities among fatigue or pruritus cohorts in the follow-up period. Proportion of patients in **(A)** the fatigue cohort and **(B)** the pruritus cohort with **(i)** anxiety, **(ii)** depression, **(iii)** UTI and **(iv)** sleep disorders over time (blue line represents case cohort and black line is control cohort). Significance level: log-rank p-value < 0.001. CI: Confidence interval; HR: Hazard ratio; IQR: Interquartile range; SD: Standard deviation; UTI: Urinary tract infection.

Certain symptoms and comorbidities were common in both the fatigue cohort and pruritus cohort during the follow-up period. Around a third (29.9%) of fatigue cases also experienced pruritus by 5-years post-index; this increased from baseline where only 9.7% of fatigue cases experienced pruritus (Table 1). The most common comorbidities among fatigue cases were anxiety (cumulative incidence rate by 5-years post-index: 55.0%), depression (47.0%), urinary tract infection (UTI; 41.7%) and sleep disorders (29.1%). Among pruritus cases, 58.0% reported fatigue by 5-years post-index which increased from the proportion reporting this symptom at baseline (24.5%). High rates of comorbid anxiety (53.7%), depression (38.2%), UTI (39.0%) and sleep disorders (31.3%) were also observed by 5-years post-index (Figure 4); information on other comorbidities is presented in Supplementary Figures 1 & 2. The risk (HR) of presenting with individual comorbidities ranged from 1.3 to 4.0 for fatigue cases relative to matched controls (all p < 0.01) and 1.5–2.8 for pruritus cases relative to matched controls (all p < 0.01) (Figure 4 & Supplementary Figures 1 & 2).

### Healthcare resource use

**Figure 5.**
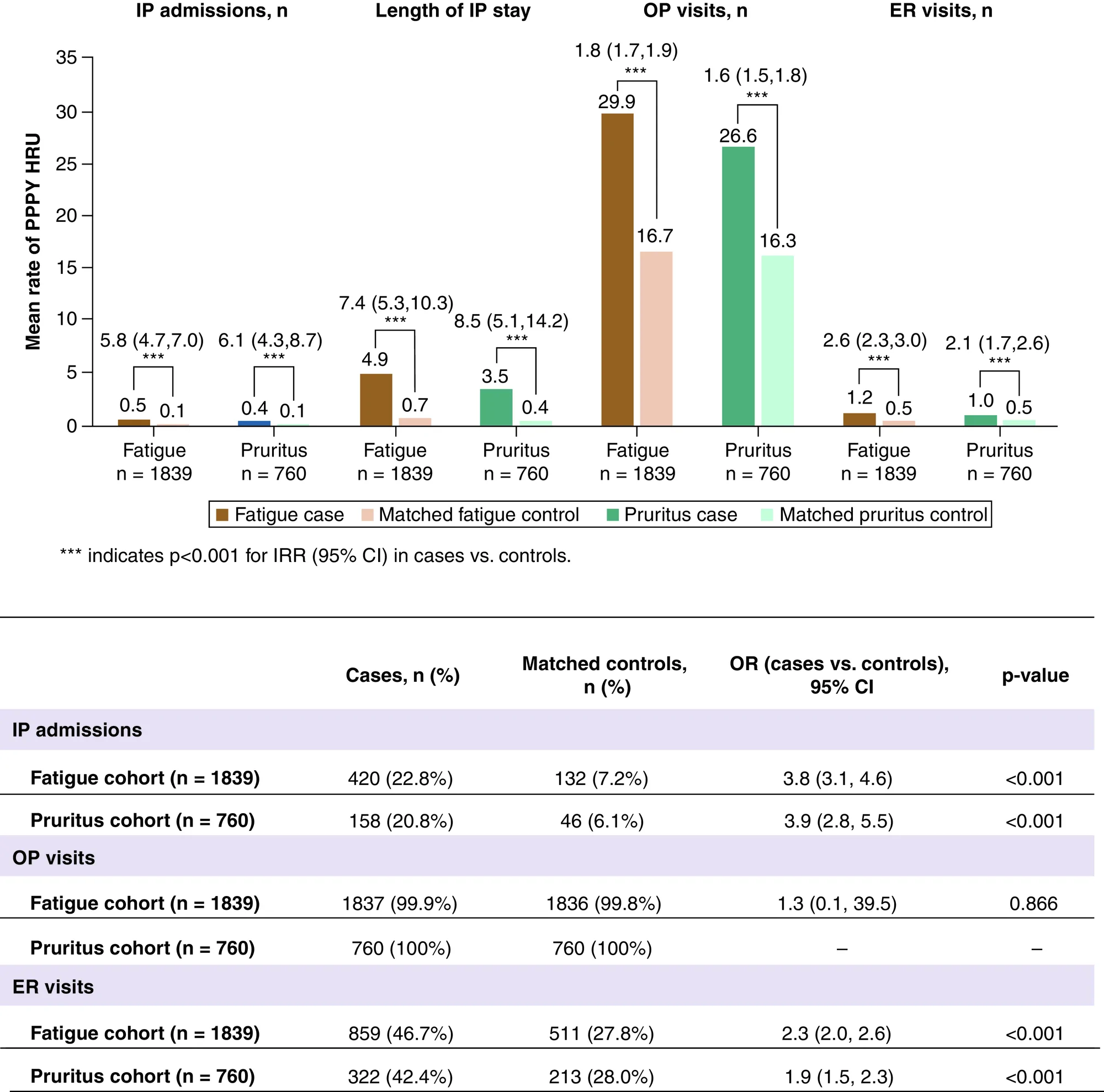
Healthcare resource use during 1-year post-index period. Mean rate of PPPY HRU for IP, OP and ER visits for the fatigue or pruritus cohorts. HRU rates were compared in cases versus controls with GEEs. IRRs (95% CI) calculated using a negative binomial distribution and log-link function are reported above the bars (*** indicates p < 0.001; significance level: p < 0.05). ORs calculated using a binomial distribution and logit-link function are reported in the table. CI: Confidence interval; ER: Emergency room; GEE: Generalized estimating equation; HRU: Healthcare resource use; IP: Inpatient; IRR: Incidence rate ratio; OP: Outpatient; OR: Odds ratio; PPPY: Per-patient-per-year.

During the 1-year post-index period, cases had significantly higher HRU compared with matched controls in both cohorts (Figure 5). Specifically, for cases vs matched controls, the mean number of IP admissions PPPY was higher for both the fatigue cohort and pruritus cohort (0.5 vs 0.1 [IRR: 5.8]; 0.4 vs 0.1 [IRR: 6.1], respectively). The mean length of IP stay PPPY was higher for both the fatigue cohort and pruritus cohort (4.9 vs 0.7 days [IRR: 7.4]; 3.5 vs 0.4 days [IRR: 8.5], respectively). This pattern was also observed for the mean number of OP visits PPPY and the mean number of ER visits PPPY (OP: 29.9 vs 16.7 [IRR: 1.8], 26.6 vs 16.3 [IRR: 1.6] for the fatigue cohort and pruritus cohort, respectively; ER: 1.2 vs 0.5 [IRR: 2.6], 1.0 vs 0.5 [IRR: 2.1], for the fatigue cohort and pruritus cohort, respectively) (all p < 0.001) (Figure 5).

A significantly greater proportion of fatigue cases or pruritus cases vs matched controls had at least one IP admission (22.8% vs 7.2% [OR: 3.8] in fatigue cohorts and 20.8% vs 6.1% [OR: 3.9] in pruritus cohorts; both p < 0.001) and at least one ER visit (46.7% vs 27.8% [OR: 2.3] in fatigue cohorts and 42.4% vs 28.0% [OR: 1.9] in pruritus cohorts; both p < 0.001) during the 1-year post-index period (Figure 5).

### Healthcare costs

**Figure 6.**
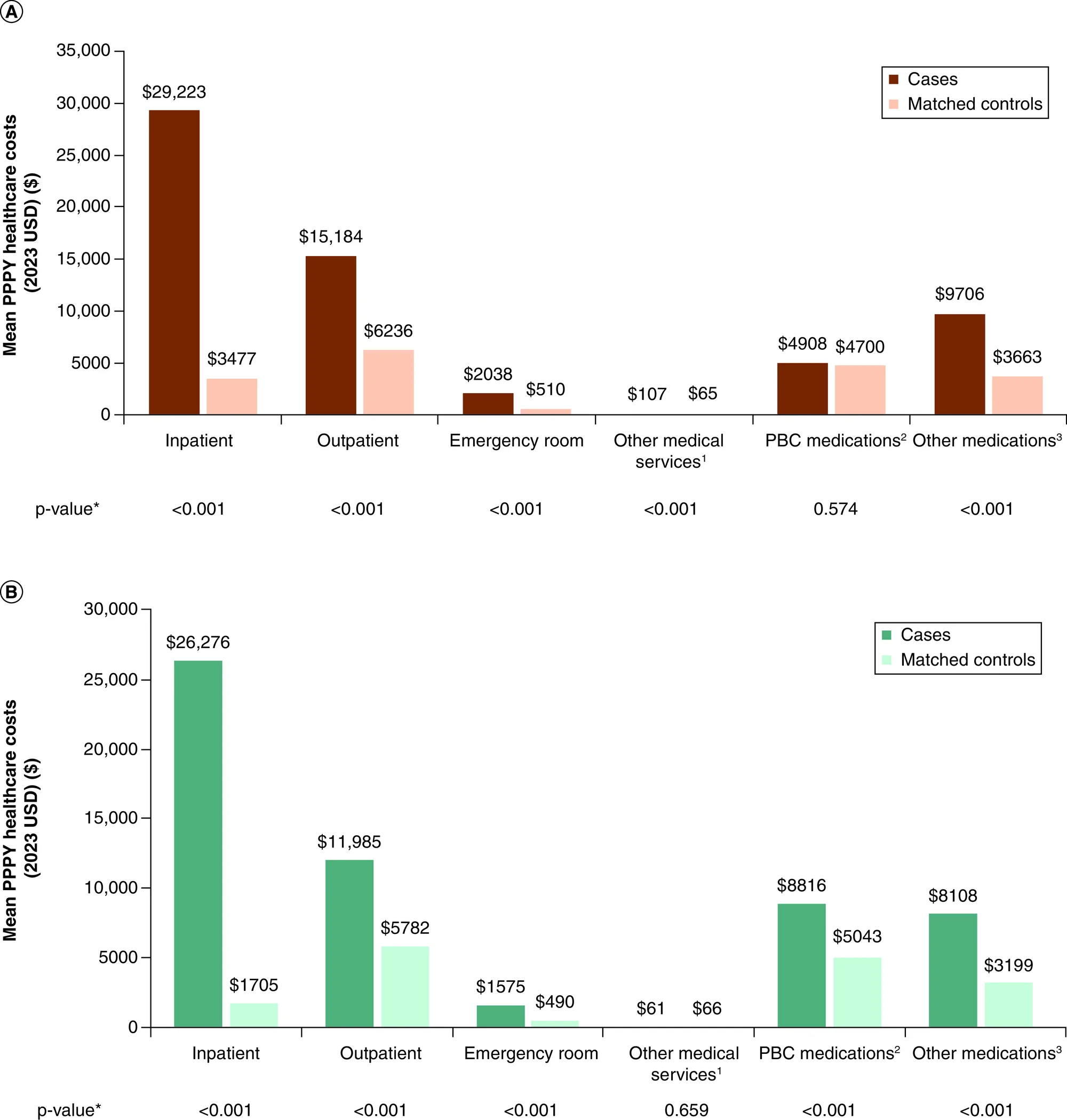
Healthcare costs during 1-year post-index period. **(A)** Fatigue cohorts. **(B)** Pruritus cohorts. Mean PPPY healthcare costs in the **(A)** fatigue or **(B)** pruritus cohorts assessed using GEE models with a Tweedie distribution and log-link function. *p-values estimated using bootstrap of 500 replicates; significance level: p < 0.05. ^1^Includes durable medical equipment and dental/visual care. ^2^Includes ursodeoxycholic acid, obeticholic acid, fenofibrate and gemfibrozil. ^3^Includes non-PBC medications; costs comprised of fatigue- or pruritus-related medications (for each cohort, respectively) and other pharmacy costs. Figure 6 component sums may differ from total costs in Results due to rounding. GEE: Generalized estimating equation; OCA: Obeticholic acid; PBC: Primary biliary cholangitis; PPPY: Per-patient-per-year; UDCA: Ursodeoxycholic acid; USD: United States dollar.

During the 1-year post-index period, cases incurred significantly higher PPPY total healthcare costs than matched controls in both the fatigue cohort and pruritus cohort ($61,167 vs $18,651 [p < 0.001] and $56,822 vs $16,286 [p < 0.001], respectively). Patients in both the fatigue cohort and pruritus cohort also had higher total medical costs compared with matched controls ($46,552 vs $10,288 [p < 0.001] and $39,897 vs $8044 [p < 0.001], respectively) and higher total pharmacy costs ($14,614 vs $8363 [p < 0.001] and $16,925 vs $8242 [p < 0.001], respectively). The largest component of medical costs for fatigue or pruritus cases versus matched controls was IP costs ($29,223 vs $3477 [p < 0.001] and $26,276 vs $1705 [p < 0.001], respectively), followed by OP costs ($15,184 vs $6236 [p < 0.001] and $11,985 vs $5782 [p < 0.001], respectively) and ER costs ($2038 vs $510 [p < 0.001] and $1575 vs $490 [p < 0.001], respectively). Costs of PBC medications were higher for fatigue or pruritus cases versus matched controls ($4908 vs $4700 [p = 0.574] and $8816 vs $5043 [p < 0.001], respectively); costs of other medications ($9706 vs $3663 [p < 0.001] and $8108 vs $3199 [p < 0.001], respectively) were also higher in cases than in matched controls (Figure 6).

The difference in total annualized costs between fatigue cases and matched controls was $42,515; the difference was $40,536 between pruritus cases and matched controls (both p < 0.001). The differences were mainly driven by IP costs ($25,746 and $24,571 difference between fatigue cases and pruritus cases vs matched controls, respectively) and OP costs ($8,949 and $6,203 difference in costs; both p < 0.001).

## Discussion

This retrospective real-world study found that patients with PBC who experienced fatigue or pruritus had a significantly higher comorbidity burden than patients without these symptoms. Notably, the comorbidity burden among patients with fatigue or pruritus increased over time following diagnosis, and at a higher rate than that of patients without these symptoms. With a median follow-up of approximately 2 years, about 50% of fatigue or pruritus cases newly reported at least three comorbidities of interest, compared with fewer than 10% in their respective matched controls. The risk of developing each comorbidity was also significantly higher in cases than in matched controls. The most common comorbidities associated with fatigue or pruritus were anxiety, UTIs, depression and sleep disorders.

The comorbidities observed in the present study generally align with those previously reported [7,20,29]. For instance, in a case-control study using data from the UK General Practice Research Database, UTI was frequently experienced by patients with PBC years before PBC diagnosis, and the risk of developing a UTI was higher among patients with PBC than among those with other chronic liver diseases [29]. In addition, high rates of anxiety and depressive symptoms have been reported by patients with PBC, and these mental health-related comorbidities have a negative impact on patients' perceived quality of life [7,30,31]. In this study, the rate of anxiety, depression, UTIs and other comorbidities typically associated with PBC was nearly double in cases vs matched controls and increased over time in both cases and matched controls [7,20,21,32]. The presence of one or more comorbidities could potentially cause a reduction in overall quality of life in patients with PBC. However, because this analysis relies on ICD codes, which confirm the presence of a symptom but do not capture information on symptom onset, duration or severity, there may be a bias toward the inclusion of more severe cases and a causal relationship between comorbidities and reduced quality of life cannot be definitively deducted from the data. The present findings highlight the importance of evaluating and managing these comorbidities in patients with PBC, especially among those experiencing fatigue or pruritus.

A previous cross-sectional study using 2016–2017 US commercial claims data found a significantly higher prevalence of comorbidities among patients with PBC experiencing pruritus compared with age- and sex-matched controls without pruritus [20]. The present study expands on the results from this earlier study by comparing a larger sample of pruritus cases with controls who were matched on several variables and by reporting the comorbidity burden at different timepoints over a longer follow-up period following the 2016 accelerated conditional approval of OCA, the first second-line treatment available for PBC. While this study cannot directly measure impact of OCA on the treatment landscape, the availability of the treatment during the study period indicates a more representative disease landscape compared with studies focusing on the period before approval of OCA. Importantly, the present study also assessed the comorbidity burden associated with fatigue, another major symptom of PBC, and included more than twice the number of fatigue cases than pruritus cases, further expanding our understanding of comorbidity burden experienced by patients with PBC.

To the best of our knowledge, this is the first study to elucidate both the incremental clinical and economic burden associated with fatigue or pruritus in patients with PBC in the US population. The present study uses recent data to demonstrate that patients with PBC who have fatigue or pruritus experience substantial incremental HRU and cost burden relative to those without these symptoms. The incremental increase in HRU was particularly evident for IP admissions, possibly due to patients with fatigue or pruritus experiencing more severe illness, which may have contributed to the increased healthcare costs. Over 12 months during the follow-up period, the total healthcare costs were three-times higher and medical costs four-times higher among fatigue or pruritus cases than their respective matched controls, underscoring the significant impact of these symptoms on the overall PBC burden. These findings are consistent with previous US studies, which have shown high healthcare costs incurred by patients with PBC [22,23].

Collectively, the findings from this study demonstrate that symptoms of fatigue or pruritus in patients with PBC are associated with a high and increasing comorbidity burden over time – with numerically higher HRU and costs among patients with fatigue or pruritus compared with matched controls in this analysis – and likely contribute to incremental increases in HRU and cost burden over time. This highlights the progressive nature of PBC, its impact on patients' lives and the unmet need for effective treatments to relieve symptom burden, particularly fatigue or pruritus.

### Strengths & limitations

As with any study, the findings should be considered with certain limitations. First, data contributors of the PharMetrics Plus database are largely commercial health plans; therefore, the results may not be generalizable to patients with PBC covered by other health plan types (e.g., Medicare and Medicaid). Second, claims-based analyses are subject to inherent limitations such as errors or omissions in diagnosis codes, inability to capture medical services or pharmacy dispensing outside of a patient's plan, and unavailability of lab test results (which may help to better understand the severity of a disease and its progression/response to treatment). In addition, the use of ICD codes alone to specify comorbidities may only capture severe events, leading to underestimation of the comorbidity burden. In PBC, patients may experience symptoms such as fatigue and/or pruritus prior to a formal diagnosis [33], potentially resulting in underestimation of symptom duration and corresponding disease burden, as medical records may be more likely to reflect comorbidities documented after symptom onset but prior to the first recorded diagnosis code.

Furthermore, claims data do not capture symptom severity or duration and are therefore unable to distinguish between mild and severe fatigue or pruritus and how symptom severity may impact comorbidity or economic burden. As many patients with PBC experience both fatigue and pruritus, the comorbidity and cost estimates are not independent, thus we are unable to determine the effects of each symptom. This was a retrospective study, which prevented establishing causal relationships between fatigue and pruritus and the increased comorbidity and economic burden.

Treatment patterns were also not included in this analysis, limiting the ability to interpret reasons for increased HRU and costs. The study duration of 12 months limits the interpretability of HRU and cost data, thus limiting the ability to draw conclusions about the long-term economic burden of fatigue or pruritus.

Nonetheless, this study is among the first to investigate the real-world cost for patients living with PBC and experiencing fatigue or pruritus in the US. Although studies analyzing medical claims data are not able to provide additional information on diagnosis and management of a disease (as no lab or medical history data are included in the analyses), the findings from these studies provide important information on the average comorbidity and economic burden of a given disease on patients (living with or without the symptoms in question). Such insights on the comorbidity and economic burden for patients living with a disease can only be captured through the analysis of medical claims data, which emphasizes the importance of incorporating real-world evidence into research to identify unmet needs and high clinical and economic burdens within a given patient population.

## Conclusion

This real-world study found substantial clinical and economic burden in patients with PBC who experienced fatigue or pruritus relative to PBC patients without these symptoms, highlighting an unmet need for effective treatments to alleviate PBC symptoms. Future studies should assess the potential impact of newer therapies on comorbidities, HRU and cost burden among real-world patients with PBC and those experiencing fatigue or pruritus. In addition, future efforts could focus on highlighting the need for closer monitoring of patients with these symptoms to promptly address and prevent escalation of future healthcare issues.

## Summary points

- Primary biliary cholangitis (PBC) is a rare, chronic, progressive and cholestatic autoimmune liver disease.
- Fatigue and pruritus are the most common symptoms of PBC, experienced by up to an estimated 75% of patients, and can have a considerable impact on patients' quality of life.
- To understand the incremental burden of fatigue or pruritus, this study evaluated the comorbidity burden, healthcare resource use (HRU) and healthcare costs associated with these symptoms among patients with PBC in the US, using claims data from the IQVIA PharMetrics^®^ Plus database (January 2016 to December 2022).
- Patients with PBC and fatigue and/or pruritus were selected as cases and matched 1:1 with controls (patients with PBC and no fatigue or pruritus) by key characteristics. After matching, 1839 fatigue cases and matched controls and 760 pruritus cases and matched controls were included in the analyses.
- The study identified that patients with pruritus experienced high rates of fatigue, and patients with fatigue experienced pruritus, albeit to a lesser degree, highlighting that a considerable proportion of patients with PBC may experience both symptoms.
- Comorbidities at 1, 3 and 5-years post-index were higher for cases than controls (fatigue: 1.7 vs 0.7, 2.2 vs 0.9 and 2.5 vs 1.0; pruritus: 1.9 vs 0.8, 2.3 vs 1.0 and 2.7 vs 1.0; all p < 0.001).
- Common comorbidities were anxiety, urinary tract infection, depression and sleep disorders (hazard ratios in cases vs controls: fatigue, 1.3–4.0; pruritus, 1.5–2.8; all p < 0.01).
- Over 12 months during the follow-up period, the total healthcare costs were three-times higher among fatigue or pruritus cases than their respective matched controls (total healthcare cost: $61,167 vs $18,651 [p < 0.001] and $56,822 vs $16,286 [p < 0.001]).
- Over 12 months during the follow-up period, the medical costs four-times higher among fatigue or pruritus cases than their respective matched controls (medical costs: $46,552 vs $10,288 [p < 0.001] and $39,897 vs $8044 [p < 0.001]).
- Collectively, the findings from this study demonstrate that symptoms of fatigue or pruritus in patients with PBC are associated with a high and increasing comorbidity burden over time, and likely contribute to incremental increases in HRU and costs.

## Supplementary Material


