# Supplementary material for: Burden of fatigue or pruritus in patients with primary biliary cholangitis: a United States matched case–control study
Source: J Comp Eff Res. 2026 Aug 12;15(9):e250179. doi: 10.57264/cer-2025-0179 (PMC13556923; doi:10.57264/cer-2025-0179)
Supplement: Supplementary file 1 [file cer-15-250179-s1.docx]

# SUPPLEMENTARY MATERIALS

FIGURES

**Supplementary Figure 1. Cumulative incidence of other comorbidities among fatigue cohorts.**

(A) **Autoimmune thyroid**

Proportion (shown as a percentage) of patients with autoimmune thyroid over time (blue line represents case cohort and black line is control cohort). Level of significance: log-rank p-value <0.001.

(B) **Sjögren’s disease**

Proportion (shown as a percentage) of patients with Sjögren’s disease over time (blue line represents case cohort and black line is control cohort). Level of significance: log-rank p-value <0.001.

(C) **Raynaud’s syndrome**

Proportion (shown as a percentage) of patients with Raynaud’s syndrome over time (blue line represents case cohort and black line is control cohort). Level of significance: log-rank p-value <0.01.

(D) **Systemic lupus erythematosus**

Proportion (shown as a percentage) of patients with systemic lupus erythematosus over time (blue line represents case cohort and black line is control cohort). Level of significance: log-rank p-value <0.001.

(E) **Rheumatoid arthritis**

Proportion (shown as a percentage) of patients with rheumatoid arthritis over time (blue line represents case cohort and black line is control cohort). Level of significance: log-rank p-value <0.001.

(F) **Cognitive impairment**

Proportion (shown as a percentage) of patients with cognitive impairment over time (blue line represents case cohort and black line is control cohort). Level of significance: log-rank p-value <0.001.

**Abbreviations**: CI, Confidence interval; HR, Hazard ratio; IQR, Interquartile range; SD, Standard deviation.

**Supplementary Figure 2. Cumulative incidence of other comorbidities among pruritus cohorts.**

(A) **Autoimmune thyroid**

Proportion (shown as a percentage) of patients with autoimmune thyroid over time (blue line represents case cohort and black line is control cohort). Level of significance: log-rank p-value <0.01.

(B) **Sjögren’s disease**

Proportion (shown as a percentage) of patients with Sjögren’s disease over time (blue line represents case cohort and black line is control cohort). Level of significance: log-rank p-value <0.001.

(C) **Raynaud’s syndrome**

Proportion (shown as a percentage) of patients with Raynaud’s syndrome over time (blue line represents case cohort and black line is control cohort). Level of significance: log-rank p-value <0.001.

(D) **Systemic lupus erythematosus**

Proportion (shown as a percentage) of patients with systemic lupus erythematosus over time (blue line represents case cohort and black line is control cohort). Level of significance: log-rank p-value <0.001.

(E) **Rheumatoid arthritis**

Proportion (shown as a percentage) of patients with rheumatoid arthritis over time (blue line represents case cohort and black line is control cohort). Level of significance: log-rank p-value <0.001.

(F) **Cognitive impairment**

Proportion (shown as a percentage) of patients with cognitive impairment over time (blue line represents case cohort and black line is control cohort). Level of significance: log-rank p-value <0.001.

**Abbreviations**: CI, Confidence interval ; HR, Hazard ratio; IQR, Interquartile range; SD, Standard deviation.

TABLE

**Supplementary Table 1. Baseline characteristics of fatigue or pruritus cohorts before matching.**

|  | **Fatigue cohorts** | | | **Pruritus cohorts** | | |
| --- | --- | --- | --- | --- | --- | --- |
|  | **Case (N=2,383)** | **Control (N=3,159)** | **SMD^1^** | **Case (N=927)** | **Control (N=3,120)** | **SMD^1^** |
| **Demographic characteristics** | | | | | | |
| **Age at index (years),**  **mean ± SD** | 56.7 ± 11.0 | 57.0 ± 10.1 | 0.021 | 54.8 ± 11.4 | 56.8 ± 10.0 | 0.193 |
| **Female, n (%)** | 2,053 (86.2%) | 2,687 (85.1%) | 0.031 | 805 (86.8%) | 2,678 (85.8%) | 0.029 |
| **Geographic region, n (%)** |  | | 0.200 |  | | 0.258 |
| South | 1,087 (45.6%) | 1,166 (36.9%) |  | 452 (48.8%) | 1,165 (37.3%) |  |
| Midwest | 601 (25.2%) | 882 (27.9%) |  | 211 (22.8%) | 866 (27.8%) |  |
| Northeast | 387 (16.2%) | 635 (20.1%) |  | 148 (16.0%) | 628 (20.1%) |  |
| West | 305 (12.8%) | 453 (14.3%) |  | 116 (12.5%) | 439 (14.1%) |  |
| Unknown | 3 (0.1%) | 23 (0.7%) |  | 0 (0.0%) | 22 (0.7%) |  |
| **Insurance type** |  | | 0.120 |  | | 0.073 |
| Commercial | 2,123 (89.1%) | 2,924 (92.6%) |  | 846 (91.3%) | 2,908 (93.2%) |  |
| Medicare Advantage | 260 (10.9%) | 235 (7.4%) |  | 81 (8.7%) | 212 (6.8%) |  |
| **PBC disease characteristics** | | | | | | |
| **First observed diagnosis,^2^ n (%)** |  | | 0.208 |  | | 0.241 |
| 2016 | 1,111 (46.6%) | 1,394 (44.1%) |  | 463 (49.9%) | 1,379 (44.2%) |  |
| 2017 | 410 (17.2%) | 482 (15.3%) |  | 157 (16.9%) | 478 (15.3%) |  |
| 2018 | 301 (12.6%) | 384 (12.2%) |  | 112 (12.1%) | 380 (12.2%) |  |
| 2019 | 297 (12.5%) | 345 (10.9%) |  | 102 (11.0%) | 340 (10.9%) |  |
| 2020 | 199 (8.4%) | 355 (11.2%) |  | 72 (7.8%) | 351 (11.3%) |  |
| 2021 | 65 (2.7%) | 199 (6.3%) |  | 21 (2.3%) | 192 (6.2%) |  |
| **PBC duration^3^ (months), mean ± SD** | 20.4 ± 16.8 | 16.2 ± 15.3 | 0.263 | 21.1 ± 16.9 | 16.0 ± 14.8 | 0.323 |
| **Comorbidities** | | | | | | |
| **CCI,^4^ mean ± SD** | 3.2 ± 1.9 | 2.6 ± 1.3 | 0.364 | 3.1 ± 1.7 | 2.6 ± 1.4 | 0.301 |
| **Comorbid liver complications, n (%)** | | | | | | |
| Abnormal liver test | 539 (22.6%) | 636 (20.1%) | 0.061 | 231 (24.9%) | 636 (20.4%) | 0.108 |
| Cirrhosis | 685 (28.7%) | 590 (18.7%) | 0.238 | 263 (28.4%) | 585 (18.8%) | 0.228 |
| Autoimmune hepatitis | 297 (12.5%) | 328 (10.4%) | 0.065 | 128 (13.8%) | 335 (10.7%) | 0.094 |
| Portal hypertension | 280 (11.7%) | 187 (5.9%) | 0.207 | 124 (13.4%) | 196 (6.3%) | 0.240 |
| Primary sclerosing cholangitis | 119 (5.0%) | 99 (3.1%) | 0.094 | 65 (7.0%) | 99 (3.2%) | 0.175 |
| Chronic hepatitis NOS | 64 (2.7%) | 96 (3.0%) | 0.021 | 28 (3.0%) | 89 (2.9%) | 0.010 |
| Liver fibrosis | 45 (1.9%) | 55 (1.7%) | 0.011 | 24 (2.6%) | 55 (1.8%) | 0.057 |
| Cholecystitis | 50 (2.1%) | 33 (1.0%) | 0.085 | 12 (1.3%) | 30 (1.0%) | 0.032 |
| **Other comorbidities, n (%)** | | | | | | |
| Pruritus | 232 (9.7%) | 0 (0.0%) | 0.464 | 332 (35.8%) | 0 (0.0%) | 1.056 |
| Fatigue | 819 (34.4%) | 0 (0.0%) | 1.023 | 220 (23.7%) | 0 (0.0%) | 0.789 |
| Dyslipidemia | 1,099 (46.1%) | 1,261 (39.9%) | 0.125 | 391 (42.2%) | 1,254 (40.2%) | 0.040 |
| Hypertension | 1,100 (46.2%) | 1,193 (37.8%) | 0.171 | 389 (42.0%) | 1,188 (38.1%) | 0.079 |
| Anxiety | 581 (24.4%) | 449 (14.2%) | 0.260 | 200 (21.6%) | 461 (14.8%) | 0.177 |
| Type 2 diabetes | 476 (20.0%) | 458 (14.5%) | 0.145 | 154 (16.6%) | 444 (14.2%) | 0.066 |
| Depression | 513 (21.5%) | 341 (10.8%) | 0.295 | 175 (18.9%) | 341 (10.9%) | 0.225 |
| Osteoporosis | 294 (12.3%) | 282 (8.9%) | 0.111 | 107 (11.5%) | 279 (8.9%) | 0.086 |
| Urinary tract infection | 371 (15.6%) | 230 (7.3%) | 0.263 | 117 (12.6%) | 228 (7.3%) | 0.178 |
| Gallstone disease (cholelithiasis) | 217 (9.1%) | 239 (7.6%) | 0.056 | 78 (8.4%) | 212 (6.8%) | 0.061 |
| Diarrhea | 317 (13.3%) | 171 (5.4%) | 0.273 | 132 (14.2%) | 152 (4.9%) | 0.323 |
| Sleep-related issues | 252 (10.6%) | 141 (4.5%) | 0.233 | 74 (8.0%) | 130 (4.2%) | 0.160 |
| Inflammatory bowel disease | 191 (8.0%) | 111 (3.5%) | 0.194 | 75 (8.1%) | 123 (3.9%) | 0.175 |
| Rheumatoid arthritis | 181 (7.6%) | 107 (3.4%) | 0.186 | 65 (7.0%) | 104 (3.3%) | 0.167 |
| Ulcerative colitis | 127 (5.3%) | 108 (3.4%) | 0.094 | 65 (7.0%) | 99 (3.2%) | 0.175 |
| Sjögren’s disease | 153 (6.4%) | 103 (3.3%) | 0.148 | 56 (6.0%) | 100 (3.2%) | 0.135 |
| Raynaud’s syndrome | 89 (3.7%) | 88 (2.8%) | 0.053 | 32 (3.5%) | 82 (2.6%) | 0.048 |
| Systemic lupus erythematosus | 83 (3.5%) | 53 (1.7%) | 0.114 | 36 (3.9%) | 49 (1.6%) | 0.142 |
| Crohn's disease | 69 (2.9%) | 50 (1.6%) | 0.089 | 32 (3.5%) | 51 (1.6%) | 0.116 |
| Cognitive impairment | 97 (4.1%) | 38 (1.2%) | 0.180 | 32 (3.5%) | 33 (1.1%) | 0.162 |
| Autoimmune thyroid disease | 73 (3.1%) | 49 (1.6%) | 0.101 | 22 (2.4%) | 42 (1.3%) | 0.076 |

**Abbreviations:** CCI, Charlson Comorbidity Index; NOS, Not otherwise specified; PBC, Primary biliary cholangitis; SD, Standard deviation; SMD, Standardized mean difference.

^1^SMD ≥0.1 was considered as imbalanced between the matched pairs.

^2^Year of first use of PBC diagnostic code post-2016.

^3^PBC duration is measured from the first observed PBC diagnosis to the index date.

^4^CCI was calculated based on Quan et al. 2011.[1]

REFERENCES

1.	Quan H, Li B, Couris CM *et al*. Updating and validating the Charlson Comorbidity Index and score for risk adjustment in hospital discharge abstracts using data from 6 countries. *Am J Epidemiol*. 173, 676–682 (2011).
